# Application of Variational Mode Decomposition and Whale Optimization Algorithm to Laser Ultrasonic Signal Denoising

**DOI:** 10.3390/s23010354

**Published:** 2022-12-29

**Authors:** Xing Mao, Quan Yang, Xiaocheng Wang, Jingdong Li

**Affiliations:** Institute of Engineering Technology, University of Science and Technology Beijing, Beijing 100083, China

**Keywords:** laser ultrasonic signal denoising, variational mode decomposition, Hausdorff distance, whale optimization algorithm

## Abstract

Laser ultrasound signal echoes are easily drowned out by the surrounding environmental noise in industrial field applications, and it is worthwhile to study methods of retaining the weak ultrasound signal during signal processing. To address this problem, this paper proposes to adopt the parameters optimized by the whale optimization algorithm to the variational mode decomposition (VMD) of laser ultrasound signals. The optimized parameters can avoid the frequency mixing and incomplete noise separation caused by the choice of artificial VMD parameters. The Hausdorff distance is applied in the process of reconstructing the signal to help accurately select the relevant modes and improve the signal-to-noise ratio. Simulation and experimental results show that the proposed method is feasible and effective compared with the other three available denoising methods.

## 1. Introduction

Laser ultrasonic technology, with its advantages of convenience and flexibility, high detection frequency, and spatial resolution, as one of the more commonly used major technical methods for non-destructive detection, can be used to detect metal grain size [1], defects [2], welding quality [3], stress [4], etc. Laser ultrasonic technology allows a laser to propagate throughout the material and extract internal information, such as changes in ultrasonic velocity and the frequency-related attenuation coefficient, as well as changes in the metal microstructure [5,6,7]. To achieve strictly non-destructive results, the thermoelastic mechanism needs to be utilized when the laser emits ultrasonic but at the same time, this method exhibits the disadvantage of low energy and a weak signal. To solve this problem, the laser phased array principle [8] can be used to convert the point-source light into a ring-shaped source [9] through a cone lens to achieve enhanced signal energy under the thermoelastic mechanism. Even so, the laser ultrasonic signal attenuates with an increasing propagation distance, and the signal is often drowned out by the noise. Therefore, it is particularly important to denoise the ultrasonic laser signal and accurately extract valid information.

Empirical mode decomposition (EMD), which has been widely used in signal processing, is a time-frequency analysis method proposed by Huang et al. [10] for non-stationary signal analysis with an adaptive nature,. However, the local characteristics of the conventional EMD may produce mode mixing. To improve this problem, Wu et al. [11] proposed a new method: ensemble EMD (EEMD), which uses white noise to superimpose the original signal so that the signal is continuous at different scales. This method can reduce the mode mixing, but the signal after denoising contains residual noise and the decomposition completion is poor.

In 2014, Dragomiretskiy et al. [12] proposed the variational mode decomposition (VMD) algorithm, which effectively eliminated the problem of EMD through extreme value envelope decomposition and decomposed the original signal into signal components with different frequencies and magnitudes. VMD can determine the number of mode decompositions for a given sequence according to the actual situation, and can adaptively match the optimal center frequency and finite bandwidth of each mode in the subsequent search and solution process to achieve the effective separation and frequency domain division of the intrinsic mode components of the signal. Due to the above advantages, VMD is widely used in signal processing methods involving bearing fault diagnosis, bioscience, earthquake monitoring, etc. Two key parameters in the VMD decomposition process need to be set: the number of mode decomposition layers *K* and the penalty factor *α*. However, artificially defined parameters can lead to many problems, including frequency mixing and incomplete noise separation. Several scholars have investigated various methods to obtain optimal parameters. Long et al. [13] utilized particle swarm optimization (PSO) to find the optimal parameters of the VMD for the removal of various interferences from the UHF PD signals. Hua et al. [14] used the grasshopper optimization algorithm (GOA) to obtain the best combination of parameters for VMD by decomposing the Lidar signal and thus achieving the denoised signal. Qi et al. [15] used the gray wolf optimization algorithm to obtain the best combination of parameters for VMD, and parallel EMD decomposes the signal to achieve the denoising of the Lidar signal.

In addition to the optimization method of optimal parameters, the signal denoising process based on VMD decomposition requires that the associated mode functions of the decomposition be reconstructed. When VMD is applied to noisy signals, the physical meaning of the modes—as pure signal, pure noise, or both—needs to be determined [16]. Li et al. [17] realized that the identification of noisy band-limited intrinsic mode functions (BLIMF) by using correlation-based thresholding will extract the modes and simulate the signal. However, the first mode still has a strong correlation with the noisy signal, low signal-to-noise scenario, leading to a poorly performing method. Liu et al. [18] developed a simple criterion based on detrended fluctuation analysis (DFA) to select correlated modes. This method can measure the long-range dependence of non-stationary time series, but it ignores the relationship with the simulated signal and lacks adaptability. Qi et al. [15] chose the first local maximum of the Pearson correlation coefficient method as the dividing line in EMD denoising. Before this maximum, the noise is the main part of the IMFs, and the rest is the main part of the signal. Xu et al. [19] calculated the correlation with the original function sequentially by gradually increasing the accumulated mode components and selected the mode component accumulated when the threshold is reached for the first time as the denoised signal. Although this method can obtain reasonable results, the threshold of the approximate entropy needs to be preset.

Given the above problems, a novel laser ultrasonic signal denoising method based on parameter-optimal VMD combined with the Hausdorff distance (HD) is proposed in this paper. First, the number of decomposition modes and the quadratic penalty factor are optimized using the whale optimization algorithm (WOA). Then, the optimal parameters are inputted to decompose the signal into modes; finally, the HD is used to select the relevant mode functions and reconstruct the signal to obtain the denoised signal. The proposed method is validated by simulating signals with five different signal-to-noise ratios and experimental laser ultrasonic signals for real lasers. The results show that the proposed WOA-VMD method achieves better performance compared with the other three denoising methods, effectively preserving the useful details while at the same time, denoising the original signal.

The main aim of this paper is to use sample entropy as the optimal fitness function of the whale optimization algorithm to obtain optimization parameters that can effectively perform variational pattern decomposition and avoid mode mixing or spurious components in order to achieve better denoising performance.

It can effectively prevent the weak signal from being annihilated by environmental noise or electromagnetic interference, facilitate the accurate extraction of the transverse echo signal of the laser ultrasound, and improve the accuracy for the subsequent research on the microstructure of metals.

The structure of this paper is as follows. Section 2 briefly introduces the principles of VMD, WOA, WOA-VMD, and HD, as well as the flow of the algorithm proposed in this paper. In Section 3, we analyze the denoising performance of WOA-VMD based on the processing results of simulated noisy signals and compare WOA-VMD with some commonly used denoising methods. In Section 4, we select an experimental signal to further analyze the denoising process and compare the performance between WOA-VMD and EMD. Section 5 is the conclusions of this paper.

## 2. Methods

In this section, we will study the laser ultrasonic signal denoising method based on the WOA for VMD. First, the basic principles of the VMD algorithm and the whale optimization algorithm are briefly introduced; second, the specific VMD based on WOA is illustrated when the combined fitness function is the sample entropy; then, the judgment of the relevant modes using HD is introduced; finally, the specific process of the algorithm proposed in this paper is shown.

### 2.1. The Principle of VMD

VMD treats the signal to be analyzed as a linear superposition of several mode components. Each band-limited intrinsic mode function (BLIMF) is defined as an amplitude-modulated frequency signal with the following expression [12]:

(1) $$u_{k}=A_{k}\left(t\right)\cos\varphi \left(t\right)$$

where *t* is time; *u_k_*(*t*) is the *k*th BLIMF; *A_k_*(*t*) is the instantaneous amplitude; and *φ*(*t*) is the signal phase.

The variational modes are constructed by calculating the one-sided spectrum of each BLIMF component using the Hilbert transform, and then estimating the central frequency [12]:

(2) $$\left\{ \begin{array}{l} \underset{\{u_{k},\omega_{k}\}}{\min}\{\sum_{k=1}^{K}{\left|\left|\partial_{t}\left[\left(\sigma \left(t\right)+\frac{j}{\pi t}\right)⊗u_{k}\left(t\right)\right]e^{-j\omega_{k}t}\right|\right|}_{2}^{2}\\ s.t.\sum_{k=1}^{K}u_{k}\left(t\right)=f\left(t\right) \end{array}\right.$$

where *k* = 1,2,…, *K*; σ(t) is the unit pulse function; *ω_k_* is the center frequency; *f*(*t*) is the input signal; ⊗ indicates the convolution operation; *∂t* denotes the partial derivative operation; and *j* is an imaginary number.

The constrained variational problem is transformed into an unconstrained variational problem by introducing penalty factors and the Lagrange multiplier operator to obtain the extended Lagrange expression [12].

(3) $$\begin{array}{l} L\left(\left\{u_{k}\right\},\left\{\omega_{k}\right\},\lambda \right):=\alpha {\sum_{k}\left\|\partial_{t}\left[\left(\delta \left(t\right)+\frac{j}{\pi t}\right)*u_{k}\left(t\right)\right]e^{-j\omega_{k}t}\right\|}_{2}^{2}\\ +{\left\|f\left(t\right)-\sum_{k}u_{k}\left(t\right)\right\|}_{2}^{2}+\langle \lambda \left(t\right),f\left(t\right)-\sum_{k}u_{k}\left(t\right)\rangle \end{array}$$

where α is the bandwidth parameter.

The center frequency and bandwidth of each component are continuously updated during the solution process until the iteration-stopping condition is satisfied [12].

(4) $$\sum_{k=1}^{K}\left({\left\|{\overset{⌢}{u}}_{k}^{n+1}-{\overset{⌢}{u}}_{k}^{n}\right\|}_{2}^{2}/{\left\|{\overset{⌢}{u}}_{k}^{n}\right\|}_{2}^{2}\right)<\phi$$

where: ${\overset{⌢}{u}}_{k}^{}$ is the expression after the kth BLIMF update and φ is the discriminative accuracy, generally taken as 10^−6^.

At the termination of the iteration, the signal frequency domain characteristics have been adaptively separated and then converted to the time domain by an inverse Fourier transform.

### 2.2. VMD Parameter Optimization Based on WOA

According to the VMD theory, the layers of decomposition *K* have a great influence on the decomposition results, while the penalty factor *α* is related to the decomposition accuracy [12]. Therefore, the determination of these two parameters is the key to improving the performance of VMD. The whale optimization algorithm (WOA) [20,21] is a new population intelligence optimization algorithm proposed by Mirjalili et al. of Griffith University, Australia, in 2016, which has the advantages of simple operation, few parameters, and a high ability to jump out of the local optimum.

#### 2.2.1. WOA

1. Encircling prey

The search range of the whale is the global solution space, and the location of the prey needs to be determined first in order to surround it. Since the location of the optimal design in the search velocity is not known, the WOA algorithm assumes that the current best candidate solution is the target prey, or close to the optimal solution. After defining the best search agent, the other search agents will try to update their positions toward the best search agent. This behavior is represented by the following Equations (5) and (6) [20].

(5) $$\vec{D}=\left|\vec{C}\cdot \vec{X^{*}}\left(t\right)-\vec{X}\left(t\right)\right|$$

(6) $$\vec{X}\left(t+1\right)=\vec{X^{*}}\left(t\right)-\vec{A}\cdot \vec{D}$$

where *t* is the current number of iterations; $\vec{A}$ and $\vec{C}$ are the coefficient vectors, $\vec{X^{*}}\left(t\right)$ denotes the best whale position vector so far, and $\vec{X}\left(t\right)$ denotes the current whale position vector.

The vectors $\vec{A}$ and $\vec{C}$ can be obtained from the following calculation [20]:

(7) $$\vec{A}=2\vec{a}\cdot \vec{r}-\vec{a}$$

(8) $$\vec{C}=2\cdot \vec{r}$$

where the value of $\vec{a}$ decreases linearly from 2 to 0, and $\vec{r}$ is a random vector in [0,1].

2. Hunting behavior

According to the hunting behavior of the humpback whale, it swims toward its prey with a spiral motion, so the mathematical model of the hunting behavior is as follows [20]:

(9) $$\vec{X}\left(t+1\right)=\vec{D^{\prime }}\cdot e^{bl}\cdot \cos\left(2\pi l\right)+\vec{X^{*}}\left(t\right)$$

where $\vec{D^{\prime }}=\vec{X^{*}}\left(t\right)-\vec{X}\left(t\right)$ indicates the distance between the whale and its prey, $\vec{X^{*}}\left(t\right)$ denotes the best position vector so far, *b* is the constant of the logarithmic spiral shape, and *l* is a random number in [−1, 1].

The humpback whale swims around its prey within a shrinking envelope while following a spiral path. To simulate this simultaneous behavior, we assume a 50% probability to choose either the shrinking envelope mechanism or the spiral model to update the whale’s position during optimization. The mathematical model is as follows [20]:

(10) $$\begin{array}{cc} \vec{X}\left(t+1\right)=\left\{ \begin{array}{l} \vec{X^{*}}\left(t\right)-\vec{A}\cdot \vec{D}\\ \vec{X^{*}}\left(t+1\right)=\vec{D^{\prime }}\cdot e^{bl}\cdot \cos(2\pi l)+\vec{X^{*}}\left(t\right) \end{array}\right. & \begin{array}{c} \mathrm{if}\;p<0.5\\ \mathrm{if}\;p>0.5 \end{array} \end{array}$$

The algorithm is set when $\left|\vec{A}\right|<1$, and the whale attacks the prey.

3. Search the prey

The mathematical model is as follows [20]:

(11) $$\vec{D}=\left|\vec{C}\cdot \vec{X_{rand}}-\vec{X}\right|$$

(12) $$\vec{X}\left(t+1\right)=\vec{X_{rand}}-\vec{A}\cdot \vec{D}$$

where $\vec{X_{rand}}$ is the randomly selected whale position vector, the algorithm is set to randomly select a searching individual. When $\left|\vec{A}\right|>1$, updating the position of other whales based on the randomly selected whale position, the whales are forced to deviate from the prey and thus find a more suitable prey, which can enhance the exploration capability of the algorithm, enabling the WOA algorithm to perform a global search.

#### 2.2.2. WOA-VMD

Sample Entropy [22] (SampEn) is a measurement of time series complexity performed by measuring the magnitude of the probability of generating new patterns in the signal; the greater the probability of generating new patterns, the greater the complexity of the sequence. The lower the value of sample entropy, the higher the sequence self-similarity is; the higher the value of sample entropy, the more complex the sample sequence is. When WOA is used to search for the optimal parameters of the VMD algorithm, the smallest sample entropy of the mode component is used as the fitness function, and the steps are as follows.

Step 1: Input the signal, set the parameter ranges of *K* and α in the VMD algorithm, and initialize the parameters in the WOA model, including population size, the maximum number of iterations, spatial dimension, and initial population individuals.

Step 2: Perform the VMD decomposition of the signal and calculate the fitness of each individual in the initial population. When the sample entropy is the smallest, the corresponding parameter is optimal.

Step 3: When $\left|\vec{A}\right|\leq 1$, the whale position corresponding to the minimum ranking entropy is selected as the target value for local exploitation, and then Equation (10) is selected to update the position of individual whales according to the magnitude of the *p*-value. When $\left|\vec{A}\right|>1$, the position of one whale is randomly selected to update the position of individual whales according to Equation (12), preserving the optimal fitness and the corresponding parameter combination.

Step 4: Keep the updated whale population position as the initial population for the new round and iterate through the cycle until the set maximum number of iterations is reached.

Step 5: Output the parameter combinations corresponding to the optimal whale individuals.

### 2.3. Hausdorff Distance Identification of Correlated Modes Reconstructs the Signal

After the VMD, the properties of the extracted modes must be determined, and the relevant modes need to be selected, which is a prerequisite for signal reconstruction. The VMD algorithm decomposes a signal from low to high frequencies. It is generally considered that the high-frequency area is the noise mode (uncorrelated mode), and the low-frequency area represents the pure signal mode (correlated mode).

The signal *x*(*t*) is decomposed by VMD to obtain a series of mode functions *u_k_*, *k* = 1, 2, …, *K*. The reconstructed signals are:

(13) $$y(t)=\sum_{i=1}^{k_{th}}u_{i}(t)$$

where *k*th denotes the index of the reconstructed area.

The probability density function (PDF) can reflect the difference between signal distributions. In this paper, we use HD as a similarity metric to distinguish between relevant and irrelevant patterns.

HD is a nonlinear operator that measures the similarity between two sets or two geometric figures [23]. The HD between two point sets *P* and *Q* is defined as follows [16]:

(14) $$\begin{array}{l} \mathrm{HD}\left(P,Q\right)=\max(D(P,Q),Q(Q,p))\\ D(P,Q)=\underset{p\in P}{\max}\underset{q\in Q}{\min}\left\|p-q\right\|\\ D(Q,P)=\underset{q\in Q}{\max}\underset{p\in P}{\min}\left\|q-p\right\| \end{array}$$

HD is sensitive to outliers and can react to the sharpness and narrowness of the PDF of the VMD pattern. To identify the relevant patterns, *x*(*t*) is decomposed into BLIMFs using VMD. The similarity index *L* is defined as follows [16].

(15) $$L\left(i\right)=distance\left[pdf(x(t)),pdf(BLIMF_{i}(t))\right]$$

The correlation pattern can be determined by the difference between two adjacent PDF distances. The larger the difference, the greater the change in similarity. The maximum difference value is defined as follows.

(16) θ=max|L(i+1)−L(i)|,i=1,2,…,K−1

Assuming that the maximum difference is generated between BLIMF_m_ and BLIMF_m+1_, the estimated signal *y*(*t*) can be obtained as follows.

(17) $$y(t)=\sum_{i=1}^{m}BLIMF_{i}$$

### 2.4. Flow of the Proposed Algorithm

**Figure 1:**
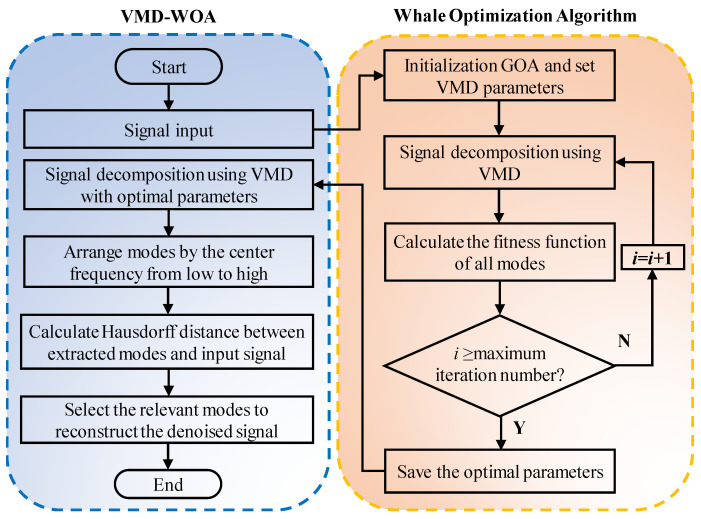
Flow chart of the proposed algorithm.

The flow of the proposed algorithm is shown in Figure 1.

First, the optimal parameters *K* and α are obtained by using the whale optimization algorithm, with the minimum sample entropy as the best fitness function.

Secondly, the optimal parameters are used as the input of VMD to decompose the signal.

Finally, using the Hausdorff distance, the relevant mode functions are selected. The relevant mode functions are accumulated to obtain the final denoised signal.

## 3. Simulations and Experiments

### 3.1. Simulation Model

**Figure 2:**
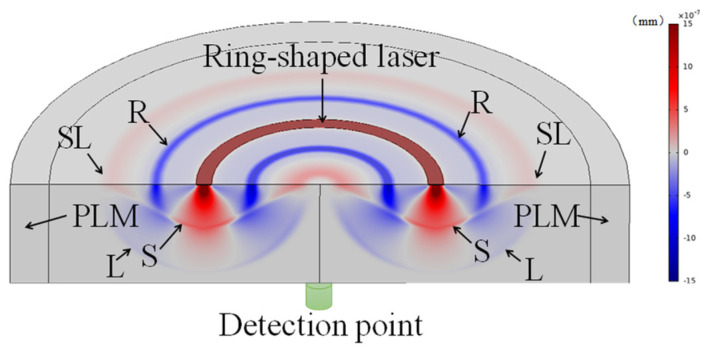
Schematic of the simulation based on COMSOL.

Laser ultrasonic simulation signals are introduced to verify the validity of the proposed method. A model of laser-induced excitation of ultrasonic fields in structural steel is developed using the commercial software COMSOL Multiphysics to simulate the generation and propagation of ultrasonic waves in the material. A solid heat transfer module is coupled to a mechanics module for the excitation of the laser ultrasonic thermoelastic mechanism, and the excitation and propagation processes of the ultrasonic waves are simulated using a transient time domain method. A two-dimensional axisymmetric model, with a radius of 8 mm and a thickness of 3 mm, is established, as shown in Figure 2. The thermal energy of a ring-shaped space is used to heat the sample to produce the ultrasonic, a detection point is set in the excitation point opposite to the lateral epicenter, and the detected displacement is used as the simulation signal of the laser ultrasonic surface waveform. The boundary is set as the perfect matched layer (PLM) to reduce the reflection from the edge. In Figure 2, S represents the shear wave, L represents the longitudinal wave, R represents the Riley wave, and SL represents the swept surface longitudinal wave.

(18) $$\Delta t=1/\left(20\times f_{\max}\right)$$

where *f*_max_ is the highest frequency of the excitation light. The grid size should be set to

(19) $$l_{e}=\lambda_{\min}/20$$

where *λ*_min_ is the shortest wavelength of excitation light.

According to the relationship between ultrasonic length and frequency, it is known that

(20) $$\lambda_{\min}=c_{L}/f_{\min}$$

where *f*_max_ is the highest ultrasonic frequency, and c*_L_* is ultrasonic longitudinal wave velocity.

In this work, the time step is set as 1 ns. The minimal mesh element near the laser irritation area and the maximum mesh element away from the laser irritation area are 5 μm and 100 μm, respectively. The material parameters used in FEM are listed in Table 1.

The noiseless laser ultrasonic signal *s*(*t*) is obtained through the above simulation. The Gaussian noise is superimposed on the laser ultrasonic signal to obtain the noisy signal *x*(*t*), as shown in the following formula:

(21) x(t)=s(t)+n(t)

where *n*(*t*) represents the generated white Gaussian noise.

To evaluate the criteria, signal to noise ratio (SNR) and root mean square error (RMSE) are adopted to compare the four denoising methods, and the expressions are as follows Equations (22) and (23).

(22) $$\mathrm{SNR}=10\log\left(\frac{\sum_{n=1}^{L}y^{2}(n)}{\sum_{n=1}^{L}{\left|y(n)-x^{*}(n)\right|}^{2}}\right)$$

(23) $$RMSE=\sqrt{\frac{1}{L}\sum_{n=1}^{L}{\left|y(n)-x^{*}(n)\right|}^{2}}$$

where *y*(*n*) is the original signal; x*(*n*) is the denoised signal; and *L* is the signal length. The higher the SNR, the better the noise reduction effect; the smaller the RMSR, the better the noise reduction effect.

### 3.2. Laser Ultrasonic Experiment

**Figure 3:**
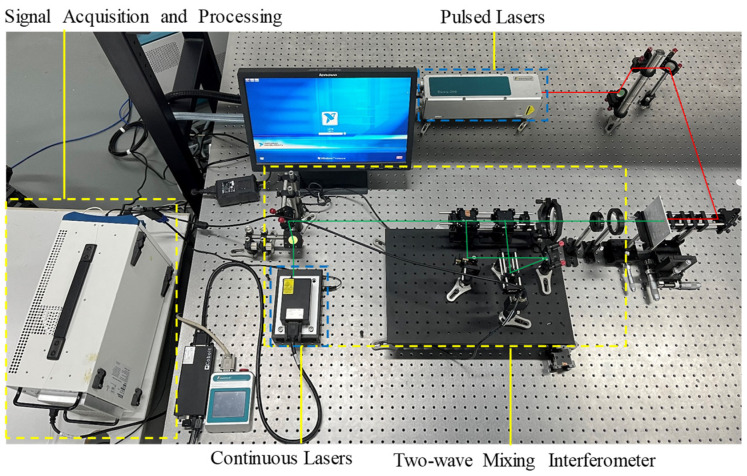
A laser ultrasonic system pulsed laser and an interferometer are used to generate and detect ultrasonic waves.

The experimental laser ultrasonic system used in this paper is shown in Figure 3. It includes an excitation system, a two-wave mixing interferometer detection system, and signal processing. A Q-tuned Nd: YAG pulsed laser is focused on the sample surface. To ensure a completely non-destructive method, the laser generates a ring-shaped laser through a conical lens, which generates ultrasonic waves to achieve maximum energy under a thermoelastic mechanism, thus enhancing the strength of the detected signal. The laser wavelength is 1064 nm and the energy of the single beam is 200 mJ. A continuous laser signal with a wavelength of 532 nm is received by a two-wave mixing interferometer that uses a continuous laser. An oscilloscope and a computer are connected to the two-wave mixing interferometer to acquire and record the ultrasonic signals. The sampling interval of the oscilloscope is 4 ns and the sampling frequency is 250 MHz. The laser parameters are shown in Table 2.

For the laser ultrasonic system shown in Figure 3, the sources of noise include, but are not limited to:

(1) Noise from the continuous laser itself.
(2) Noise from the photodetector.
(3) Electromagnetic interference generated by the excitation of the pulsed laser.

## 4. Results and Discussion

### 4.1. Simulation Signal

**Figure 4:**
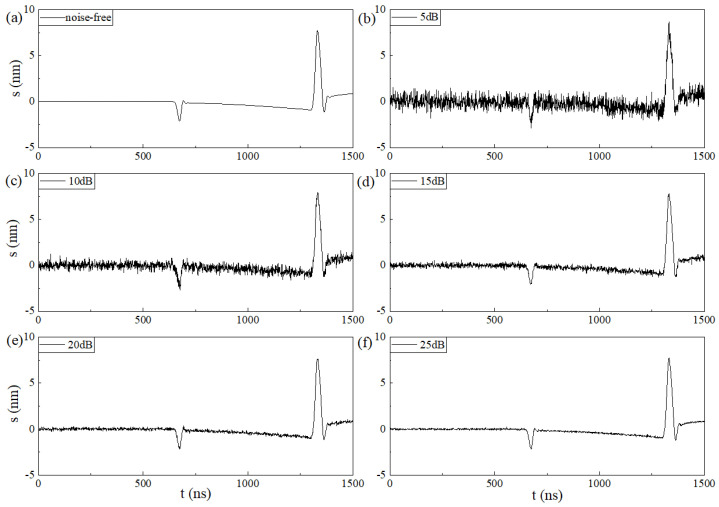
The simulation signal. (**a**) Noise-free signal. (**b**) SNR_in_ = 5 dB. (**c**) SNR_in_ = 10 dB. (**d**) SNR_in_ = 15 dB. (**e**) SNR_in_ = 20 dB. (**f**) SNR_in_ = 25 dB.

To verify the noise reduction performance of each denoising algorithm under different noise intensities, the proposed method (WOA-VMD) is compared with the other three methods: EMD, WT-db45, and singular value decomposition (SVD). To compare different signals with different levels of noise, the Gaussian noise, for which the SNR is 5 dB, 10 dB, 15 dB, 20 dB, and 25 dB, respectively, is superimposed on the laser ultrasonic signal to obtain the noisy signal. The results are shown in Figure 4.

**Figure 5:**
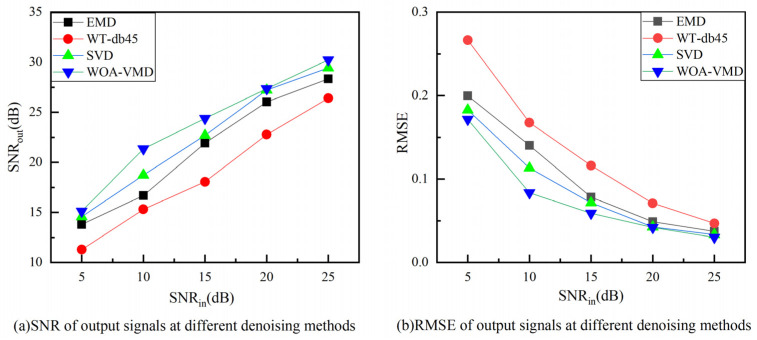
Comparison of the performance of different denoising methods in regards to different noisy signals.

The results of the denoised signal comparisons are shown in Figure 5. It can be seen that as SNR_in_ increases, the SNRs of the denoised signal with four algorithms all increase. The WT-db45 denoised signal has the lowest SNR_out_ and highest RMSE among all methods. The proposed WOA-VMD method obtains a better SNR_out_ and RMSE than the other methods for the noisy laser ultrasonic signal. When SNR_in_ = 25 dB, the signal that is processed by the proposed method achieved the highest SNR_out_ of 30.23 dB, and the corresponding RMSE is the lowest at 0.03. Even in the low SNR scenarios of SNR_in_ = 5 dB, the SNR_out_ is still 15.11 dB. The RMSE is the lowest at 0.1716.

**Figure 6:**
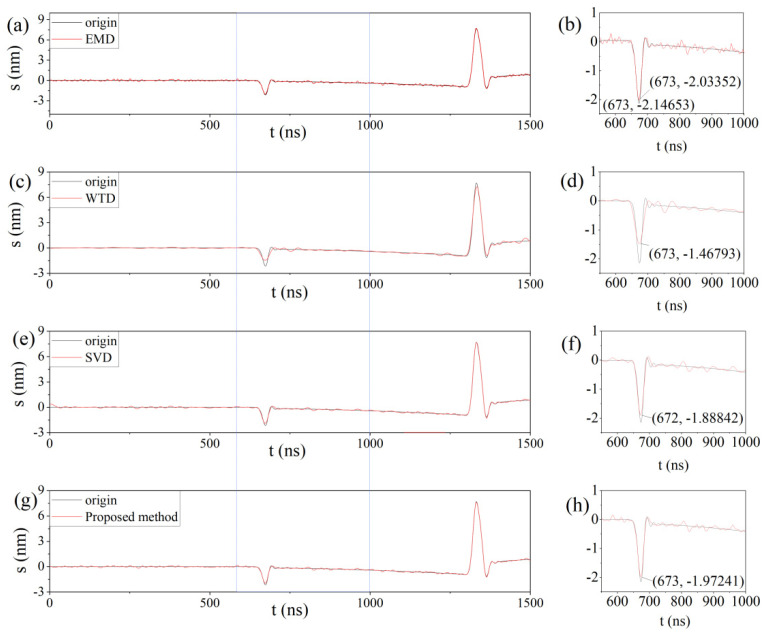
The comparison results of different denoising methods. (**a**,**c**,**e**,**g**) represents the original signal and the signal after signal processing by various methods respectively; (**b**,**d**,**f**,**h**) are the corresponding partial enlargements.

To further compare the details after denoising by different denoising methods, the comparisons before and after denoising for a signal with SNR_in_ = 15 are shown in Figure 6. The black curve in the figure shows the original signal, and the red curve shows the signal after denoising by each method. The right (b), (d), (f), and (h) correspond to local enlargements of (a), (c), (e), and (g) on the left, respectively, where the peak coordinates of the original signal and the signal after denoising by various denoising methods are identified. The peak coordinate value of the original signal is (673, −2.14653) in (b), and the peaks of the EMD, WT-db45, SVD, and WOA-VMD after denoising are (673, −2.203352) in (b), (673, −1.46793) in (d), (672, −1.88842) in (f), and (672, −1.1.97241) in (h), respectively. It can be seen that the peak value of the signal after EMD denoising is higher than that of the WOA-VMD; the signal amplitude after WTD denoising is much smaller, and the denoised signal has a larger deviation from the original signal, while the peak value of the signal after SVD denoising is shifted in the horizontal coordinates, and the peak value is lower. However, the SNR after EMD denoising is 21.9253 dB, which is lower than that of WOA-VMD, as shown in Table 3. From Figure 6a,b, it can also be seen that the denoised signal exhibits more burrs, and the signal is not smooth enough, while the WOA-VMD is smoother after denoising. Overall, the proposed method ensures the high SNR of the denoised signal, while the peak characteristics of the original signal are relatively well maintained. The signal indexes of the signals denoised by different methods when SNR_in_ = 15 dB are shown in Table 3.

### 4.2. Physics Experiments Signal

**Figure 7:**
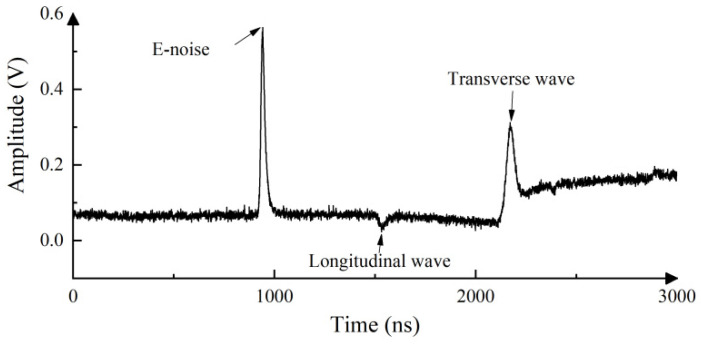
Laser ultrasonic experimental signal.

In this section, we evaluate the performance of WOA-VMD for the denoising of real laser ultrasonic signals obtained from the experiments. EMD and WOA-VMD are performed on the real laser ultrasonic signal separately to compare the decomposition performance. The actual laser ultrasonic experiment is shown in Figure 3. The ring-shaped laser diameter is set to 5 mm, and the thickness of the steel plate is about 3 mm. The obtained laser ultrasonic experimental signal is shown in Figure 7.

**Figure 8:**
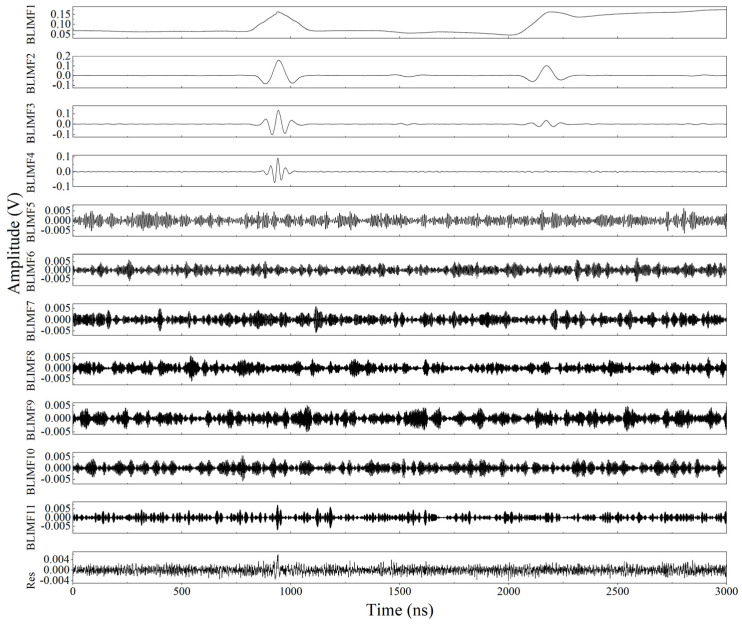
BLIMFs decomposed by the VMD method for the ultrasonic signal.

**Figure 9:**
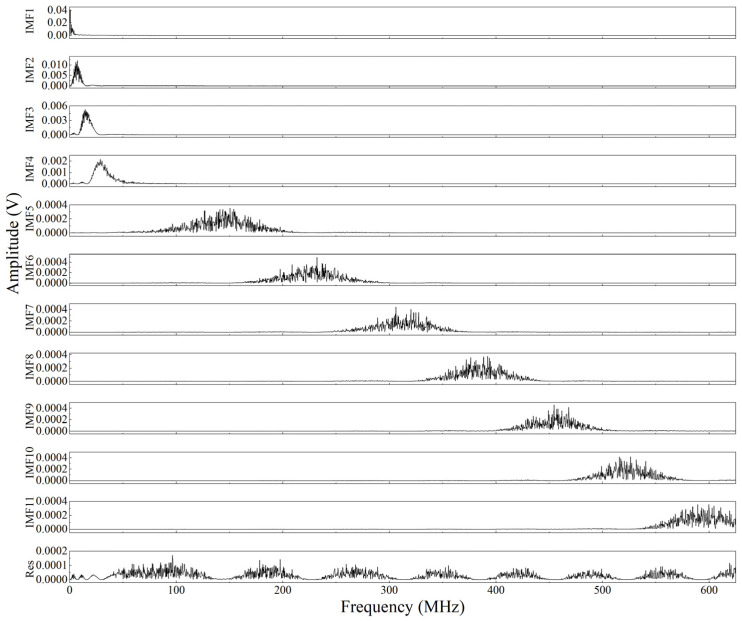
Fourier transform of the BLIMFs decomposed by the VMD method for the ultrasonic signal.

The WOA is used to process the obtained laser ultrasonic experimental signal to obtain the optimal decomposition parameters of VMD, and the parameter ranges of *K* and *α* are set from 3 to 12 and 100 to 3000, respectively. The final search result *K* value is taken as 11, and the α value is 906.8736. The obtained search result is used as the VMD decomposition parameter, and the convergence criterion tolerance is set as 1 × 10^−6^. The final VMD decomposition results and the corresponding spectral functions are shown in Figure 8 and Figure 9. It can be seen in Figure 8 that the valid components of the laser ultrasonic signal are mainly concentrated in the first few modes decomposed by the VMD. In addition, as shown in Figure 9, the major frequencies of the modes decomposed by VMD are arranged in ascending order, which indicates that the major frequencies of the laser-generated ultrasonic signal are located at low frequencies.

**Figure 10:**
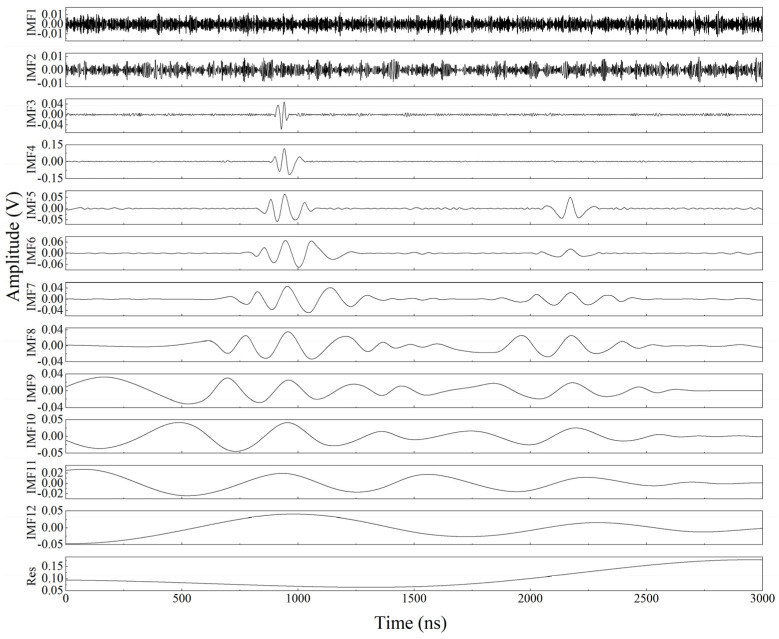
IMFs decomposed by the EMD method for the ultrasonic signal.

**Figure 11:**
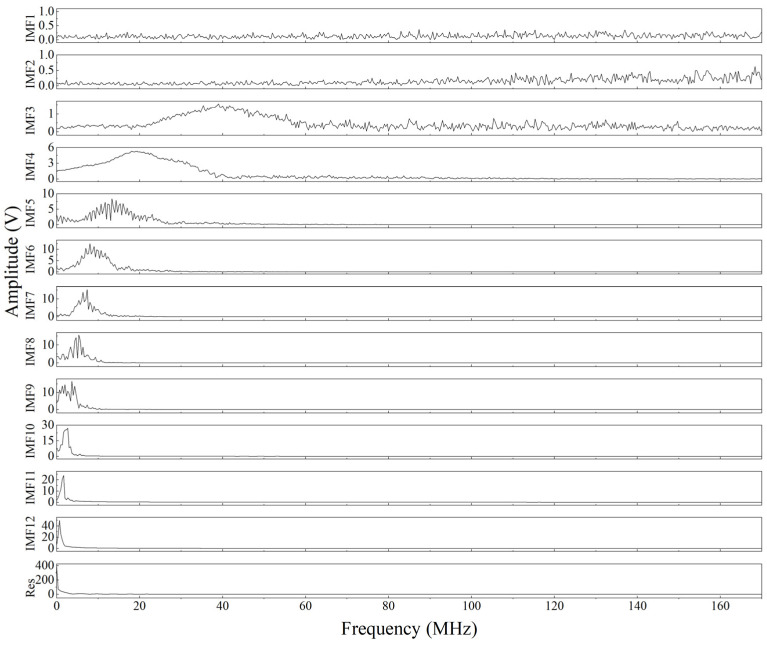
Fourier transform of the IMFs decomposed by the EMD method for the ultrasonic signal.

To better compare the effect of VMD denoising, the same signal is decomposed by EMD to obtain the results shown in Figure 10. It can be seen that the major frequencies of the IMF decomposed by EMD are arranged in descending order, and the effective components are located at the lower frequencies. As shown in Figure 10 and Figure 11, the major frequencies of the IMF decomposed by EMD are arranged in descending order, and the effective components are in the later IMFs. In addition, the frequency distribution of the IMFs indicates that EMD will result in the mode mixing of the decomposed IMFs, especially at low frequencies. In the IMF3 in Figure 11, we can see that there are both low-frequency ultrasound signals and high-frequency noise signals. However, the frequency spectrum of the modes decomposed by VMD is shown in Figure 9, and the mode spectra decomposed by VMD are separated from each other. That is, VMD can effectively reduce the mode mixing of laser ultrasonic signals.

**Figure 12:**
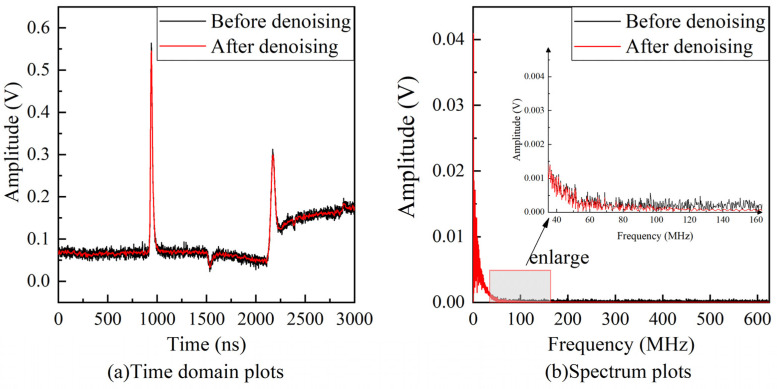
Comparison of the experimental signal before and after denoising.

The signal after decomposing the VMD, using the Hausdorff distance, the distance between the 4th and 5th components is the largest, so the first four components are chosen to be cumulatively reconstructed to obtain the final denoised signal. The comparison of the signals before and after denoising is shown in Figure 12. It can be seen that in the time domain, the signal waveform oscillation with noise becomes smooth by the method proposed in this paper. In the frequency domain, Figure 12b shows that most of the low-frequency part of the spectrum is retained, i.e., the useful information of the signal is also retained. It shows that the proposed denoising method can remove the noise in the high-frequency part while retaining the useful information in the signal, exhibiting a good denoising performance.

## 5. Conclusions

In this paper, a novel denoising method, called the WOA-VMD, is proposed to deal with the laser ultrasonic signal denoising problem.

(1) The proposed method utilizes the WOA to optimize the VMD parameters, including the number of decomposition modes *K* and penalty factor *α*. Then, relevant modes are selected using the HD between the decomposed modes and the original signal. The denoised signal is obtained by accumulating the correlation modes.

(2) The feasibility and effectiveness of this proposed method are verified via simulations. The simulation results of the ultrasonic signals with different SNR_in_ have shown that the proposed method can effectively denoise the noisy signal and outperform other denoising methods.

(3) Finally, a laser ultrasonic experiment is carried out. The results prove that the proposed method could reduce the Gaussian white noise and high-frequency noise, preserve the useful signal, and increase the SNR_out_ for laser ultrasonic signals. This helps to extract the transverse signal echoes of laser ultrasound and improves the accuracy of subsequent studies of the microstructure of metals.

## Figures and Tables

**Table 1:** Material properties of the steel model.

Property	Value	Unit
Thermal expansion coefficient α	12.3 × 10^−6^	K^−1^
Thermal conductivity k	44.5	W∙m^−1^∙K^−1^
Heat capacity C	475	J∙kg^−1^∙K^−1^
Density ρ	7850	Kg∙m^−3^

**Table 2:** Laser parameters.

Continuous Lasers		Pulsed Lasers	
Wavelength (nm)	532 ± 1	Wavelength (nm)	1064
Operation mode	Continuous	Pulse Width (ns)	≤8
Output power (w)	0.8	Single pulse energy (mJ)	200
Line width (nm)	<0.00001	Pulse frequency (Hz)	1~20
Power fluctuation range (%)	2	Power fluctuation range (%)	1
Divergence angle (mrad)	<1.5	Divergence angle (mrad)	1
Light spot diameter (1/e2, mm)	~1.5	Light spot diameter (mm)	6

**Table 3:** Comparison results of denoising indicators when SNRin = 15 dB.

Indicators	EMD	WT-db45	SVD	WOA-VMD
SNR/dB	21.9253	18.0577	22.7047	**24.3957**
RMSE/(*10^−9^)	0.0783	0.1161	0.0716	**0.0589**

## Data Availability

Not applicable.
